# Prevalence and characteristics of aquagenic pruritus in a young African population

**DOI:** 10.1186/1471-5945-9-4

**Published:** 2009-04-17

**Authors:** TA Salami, SO Samuel, KC Eze, E Irekpita, E Oziegbe, MO Momoh

**Affiliations:** 1 Department of Medicine, Faculty of Clinical Sciences, College of Medicine, Ambrose Alli University Ekpoma, Edo State, Nigeria; 2 Department of Medical Microbiology, Faculty of Clinical Sciences, College of Medicine, Ambrose Alli University Ekpoma, Edo State, Nigeria; 3 Department of Radiology, Faculty of Clinical Sciences, College of Medicine, Ambrose Alli University Ekpoma, Edo State, Nigeria; 4 Department of Surgery, Faculty of Clinical Sciences, College of Medicine, Ambrose Alli University Ekpoma, Edo State, Nigeria; 5 Department of Obstetrics and Gyneacology, Faculty of Clinical Sciences, College of Medicine, Ambrose Alli University Ekpoma, Edo State, Nigeria

## Abstract

**Background:**

Aquagenic pruritus (AP) occurs during or after contact of the skin with water such as occurs in bathing.

**Methods:**

This study aims to describe the prevalence of aquagenic pruritus in a young adult population and describe the circumstances of bathing.

A Population-based cross sectional study involving administration of Questionnaires to young adult Nigerians on the occurrence of pruritus associated with bathing.

**Results:**

The prevalence of bathing pruritus among respondents in this study was 23.8%. The commonest type of water respondents itch to was rain water (23%) followed by cold water (19%). 8.33% of respondents feels like avoiding bathing because of these.

**Conclusion:**

Bathing pruritus is a common finding among young adult Nigerians in the general population.

## Background

Among cutaneous sensations, itching has a place of unique importance in dermatology and is still best defined as it has been for centuries as an unpleasant sensation which provokes the desire to scratch[1].

The entire skin and palpebral conjunctiva has the capacity to itch, although there are great regional and individual differences in itch sensitivity. The perianal, perigenital areas, ear canals, eyelids, and nostrils are especially prone to itching. The peripheral receptors of itching are chiefly confined to the superficially situated papillary dermis which in most areas lies within 0.2 mm of the skin surface. In all likelihood, the very superficial nerve fibre endings and network which function as receptors for itching are the same elements which function in the perception of superficial cutaneous pain.

When pruritus occurs during or after contact with water such as occurs in bathing, it is called bathing pruritus which is a form of aquagenic pruritus. This is an extremely common cause of generalized pruritus that is characterized by the development of severe, prickling like, tingling or burning skin lesion and is evoked by contact with water [2].

Various properties of water are known to be related for its ability to cause itching when used in bathing. These include the water temperature, physical characteristic/ionic content and the Ph of the water, the soap used in bathing, sponging and towelling afterwards.

Most cases are idiopathic and related only to the water itself. It has however been linked to several conditions such as juvenile xanthogranuloma [3], myelodysplastic syndrome [4], polycythaemia rubra vera [5], acute leucocytoclastic vasculitis [6], lymphoblastic leukaemia [7], T-cell non Hodgkins's lymphoma [8], metastatic squamous cell carcinoma [9], hepatitis C infection [10], drugs like bupropion [11], and antimalarials when used to treat lupus erythematosus [12], idiopathic hypereosinophilic syndrome[13].

It can be a very distressing condition which because of its relatively short duration, most people tend to ignore but it can exert psychological effect, to the extent of abandoning bathing or developing phobia to bathing.

Treatment of this condition is non specific and highly unsatisfactory in most patients perhaps because the pathogenetic and pathophysiologic mechanisms leading to it are still poorly understood. According to Greaves et al, pharmacological studies has shown that aquagenic pruritus is associated with local release of acetyl choline in the skin, mast-cell degranulation, and raised blood histamine concentrations[14] as well as to increased cutaneous fibrinolytic activity both before and after contact with water which could explain the lack of wheal formation in this condition[15].

A wide variety of both physical and pharmacologic agents such as Naltrexone [16], propanolol [17], astemizole [18], recombinant interferon alpha [19], nitroglycerin [20], topical capsaicin [21], and intramuscular triamcinolone acetonide [22] have been used as therapeutic agents with various degrees of success.

There have been frequent complaints of pruritus after bathing among young adults in this environment which oftentimes history, physical examination and investigations are not revealing of any pathology. Olumide and Oresanya had found a prevalence of bathing pruritus of 21% amongst dermatologic patients seen at the Lagos University Teaching Hospital Skin Clinic, in patients presenting with generalized pruritus without obvious skin disease [23]. However no study has been conducted to find the prevalence in the general population not presenting to the hospital to seek treatment. This is the main reason where this study was conceptualized.

This study aims to determine the prevalence of bathing pruritus in young adult Nigerians and to determine the type, characteristic and the way of use of bathing water in the production of bathing pruritus.

## Methods

This is a population based cross sectional study.

### Subjects

A total of 2000 questionnaires were administered to undergraduates of a tertiary institution-Ambrose Alli University Ekpoma, Edo State, Nigeria. Ethical approval was waived for the study because the research involved answering of questionnaires to adults who complied of their own free will and does not involve taking any body fluid specimens of any type. The university has an average population of ten thousand students distributed over ten faculties (Agriculture, Arts, Basic medical sciences, Clinical sciences, Education, Engineering and technology, Environmental studies, Law, Natural sciences, and Social sciences). The bulk of the students are not resident within the university premises due to limited accommodation facility available on campus. In this rural part of Nigeria, water is relatively scarce and water from different sources is used for daily activities. Water is mainly bought from water vending vehicles or water tankers who fetch waters from rivers or commercial borehole operators and now distribute to other commercial operators who store in big containers. During the raining season, most people will gather rain water and store for use later. These can be stored in underground 'wells' and used after the rains have stopped for several months. Another major source of potable water is directly from commercial borehole operators who sell water to people in plastic containers. In other words, the sources of water are highly varied with pipe borne water non existent, making this community an ideal place to study this kind of phenomenon with skin contact with various sources of water.

### Materials

**Figure 1:**
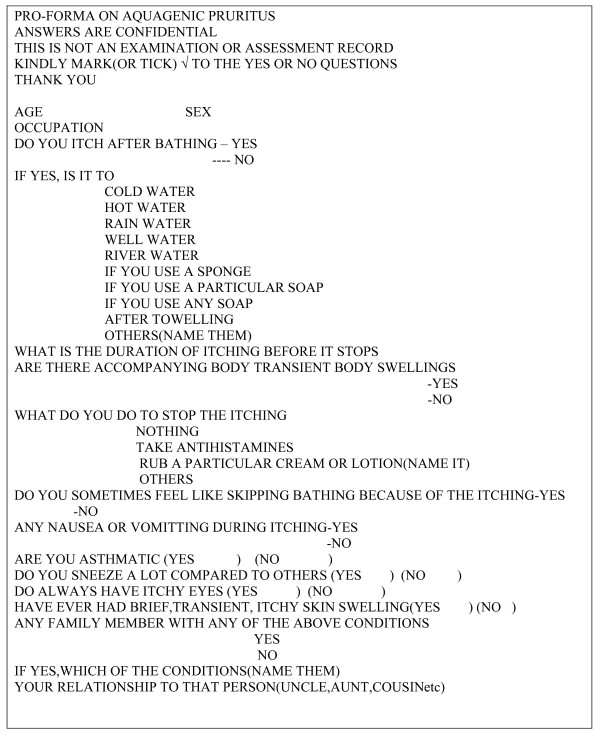
Aquagenic pruritus questionnaire

A previously designed self administered questionnaire was pretested (during a pilot study) and noted deficiencies were corrected before final administration- see figure 1. Based on the available faculties in the university, 200 questionnaires were administered to each faculty. The students were randomly selected in the faculty. Information was obtained in a standardized way with a structured self administered questionnaire since the target population is educated and most of the responses required were closed ended (based on responses obtained while pre-testing the questionnaires). The questionnaires were administered in between formal lectures on campus and then collected back immediately if possible after completing it (since the average time it took to complete the one page questionnaire is less than 15 minutes from the earlier pilot study done).

### Data analysis

Frequency distributions of some variables were determined. Independent and dependent variables were cross tabulated to examine the associations. Data was extracted and subjected to simple statistical analysis using the Microsoft Excel software.

## Results

### 1. Demographic background

A total of 2000 questionnaires were administered out of which 840 were returned. The response rate was 42% because some participants requested for more time to go through the questionnaires at a more leisurely time and were to return it later. These are the major group of respondents that did not return the completed questionnaires despite initial explanations and assurances of confidentiality.

The mean age of the respondents was 25 ± 3.8 years. Male: Female ratio 1:1.

### 2. Prevalence of reported aquagenic itch

600 respondents have never experienced bathing pruritus before while 240 has had it, giving a prevalence of 23.8%. Of these, 150(62.5%) were males while 90(37.5%) were females. Male: Female ratio 1.7:1.

The average duration of itching after bathing was 5.5 ± 2.7 minutes.

### 3. Circumstances of bathing pruritus

The breakdown of the various precipitating events is seen in Table 1 (See in the table section after the references).

**Table 1:** The breakdown of the various precipitating events as seen below in

Characteristic of water	Frequency(Males)	Frequency(females)	Both(males & females)
Cold water	25(16.7%)	20(22%)	45(19%)
Tepid water	0	5(6%)	5(2%)
Rain water	25(16.7%)	30(33)	55(23%)
Well water	30(20%)	10(11%)	40(17%)
River water	5(3.3%)	10(11%)	15(6%)
Water & sponging	30(20%)	10(11%)	40(17%)
Water & particular soap	5(3.3%)	5(6%)	10(4%)
Water & any soap	5(3.3%)	0	5(2%)
After towelling	25(16.7%)	0	25(5%)

Total	150(100%)	90(100%)	240(100%)

41/150(27%) of the males had bathing pruritus to multiple factors compared to 35/90(39%) of females. Only 20/240(8.33%) of the respondents had accompanying body swellings (aquagenic urticaria 2 (2.18%) of females, 18(12%) of males).

### 4. Interventions to mediate event

15 (6.25%) of subjects take antihistamines for the itching while 25(10.42%) indulge in activities like immediate cream application after bathing, and immediate wearing of clothes to abort the itching while the remaining 200 (83.33%) of the subjects did nothing.

### 5. Personal atopic tendencies in respondents

Ten (4%) of the subjects were asthmatic, 30(12.5%) had allergic rhinitis, 45(18.75%) had allergic conjunctivitis.

### 6. Family atopic tendencies in respondents

Seventy five (31.25%) of the subjects had family members with various atopic conditions compared to 100(16.67%) of the subjects without bathing pruritus (with bronchial asthma being the most common atopic condition-40 (40%).

### 7. Tendency to avoid bathing due to itching

20(8.33%) feels like skipping bathing because of the itching.

## Discussion

The prevalence of bathing pruritus (BP) in this study is 23.5%. This is comparable to the 21% found by Olumide and Oresanya amongst dermatologic patients seen at the Lagos University Teaching Hospital Skin Clinic, in patients presenting with generalized pruritus without obvious skin disease [23]. It is however in contrast to a prevalence of 4.5% of aquagenic pruritus found by Potasman et al in a study of 996 hospital employees in Isreal [24]. This maybe due to different backgrounds in which the studies were conducted and perhaps because classic aquagenic pruritus is a rarer entity which occurs when patients come in contact with water irrespective of its physical attributes whereas bathing pruritus is a variant that occurs more commonly during bathing but is a hardly acknowledged one.

The mean duration of itching in this study is 5.5 ± 2.7 minutes after bathing. This differs from an average of, 40.6 minutes found amongst patients with aquagenic pruritus studied by Steinman and Greaves [25]. It is however similar to the time observed by Potasman et al [24] in their patients in which the onset of itching occurred within 5 minutes of exposure in 76% of the cases and usually lasted between 10 and 30 minutes. This seems to confirm the assumption that bathing pruritus is merely a variant of the classic aquagenic pruritus and it may be a valid assumption that it can progress in that direction too.

The commonest source of water to which respondents itch was to rain water (23%) followed by cold water (19%) and well water (17%). This may be due to the relatively low pH of rain water compared to other sources of water. This theory may be responsible for the use of sodium bicarbonate baths as a way of treating aquagenic pruritus. Wolf et al reported a great success in one of their patients treated with sodium bicarbonate bath [26]. However not all patients will respond to this type of treatment as reported by Dannaker et al [27]. Well water in this study can also be equated to rain water in this study because the practice in this environment is to store rain water in underground storages called wells which can be used for some period after the raining season.

Cold water used for bathing accounted for the next commonest attribute of water responsible for itching. This is in contrast to what was observed by Potasman et al [24] in their subjects in which there was no association of water temperature with occurrence of pruritus. This is not totally unexpected as explained above.

Cold is a widely recognised cause of degranulation of mast cells whether in the skin or lungs or elsewhere which may be a strong factor in this condition. In this study 31.25% of the respondents with bathing pruritus had one type of atopic condition or the other which predisposes them to mast cell instability and degranulation compared to 16.62% of subjects not so affected. This is also notably in contrast to what was found by Steinman and Greaves [25] in their study of 36 patients with aquagenic pruritus in which there was no increased prevalence to atopy among their subjects although thirty-three percent of their patients reported a family history of water-related itching. This maybe due differences in sample selection because the subjects in the above quoted study were patients reporting in the hospital with complaints of classic aquagenic pruritus while our respondents are only responding to a pre tested questionnaire on bathing related pruritus. However it is worthy of note that heating the skin to 41°C blocks itch but increases pain perception. Also 10.42% of the affected subjects in this study try to generate body heat by immediately wearing clothes after bathing, or by moving around or immediate application of occluding creams. All this tends to support warmth or elevation of temperature stabilizing mast cells and alleviating the itching of bathing pruritus [28,29]. This is also supported by the use of PUVA [30] and PUV-B [31] therapies as a means of treatment in various reports, as well as other modalities that will generate heat such as capsaicin [21], or even alcohol [32] in the treatment of aquagenic pruritus.

Sponging and towelling are physical components of the bathing act that also tend to provoke itching in 17% of respondents (after sponging) and 5% (after towelling) respectively. Traditional African sponges consist of a tough collection of the shredded bark of trees which can inflict injury on the skin if used too vigorously. Modern sponge consist of a tough net like nylon material that is unlikely to injure like plant sponge but vigorous scouring of the body will also likely lead to activation and release of peptides as well as cutaneous mast-cell degranulation, and raised blood histamine concentrations [14]. All these will be greatly aided by sponging of the skin during bathing as widely practiced in our environment where the believe is that the more vigorously the skin is scrubbed the cleaner the individual after bathing. Thus it seems that this type of itching might be reduced by changing to a soft foam sponge or even eliminating sponging completely. Also gentle drying of the skin by patting down rather than rubbing might reduce the occurrence of itching after bathing.

A small but significant percentage of the respondents (8.33%) had a genuine phobia for bathing because of this condition. This can be understandable especially when affected individuals have tried various methods to alleviate the itching. Bathing pruritus like aquagenic pruritus is a poorly understood condition in which treatment is also very unsatisfactory unless some of these attributes of bathing are identified and avoided. Though not a life threatening condition like aquagenic urticaria and angioedema (AU), it can still exert a marked psychological effect on the affected. Its relation to AU in which there is accompanying transient body swelling is also important (8.33% of the subjects in this study responded to having transient body swellings). These wheal formations often worsen the phobia for water and the act of bathing and raise concern for both the doctor and affected individuals that seek advice. Treatment of cases such as these can be troublesome as reported by Frances et al in their case report of a patient with aquagenic urticaria [33]. A similar case of aquagenic angioedema was also reported by Parks and Camisa which occurred in a patient while swimming [34].

### Limitations of the study

The limitations of the study include

1. The study population involved mainly young adults hence generalization to other age groups in the community may not be appropriate.

2. Subjects unwilling to participate in the survey by not returning completed questionnaires are a major source of the low response rate of 42%. However, this was partially addressed by administering the questionnaires to a large number of fairly non homogenous group of participants which is still fairly representative of the study population.

3. Since no blood sample was collected from any of the participants, investigation of pathologic causes of itching cannot be pursued. This might possibly yield additional information among participants that actually itch to water. This is an area of planned future follow up research.

## Conclusion

In conclusion, bathing pruritus which is a variant of aquagenic pruritus appears to be a common condition in this environment. There are however differences in its presentatation compared to what obtains in the classic aquagenic pruritus and the rarer aquagenic urticaria and angioedema. These clinical conditions can make the act of bathing a daunting one and efforts should be made to understand these conditions in order to offer succour to patients or individuals so affected.

## Competing interests

The authors declare that they have no competing interests' Financial or otherwise. No financial aid or grant was sourced or collected from any individual or group or company. Funding for the logistics of making and administering of questionnaires was by the joint contributions of the authors.

In the past five years we have not received reimbursements, fees, funding, or salary from any organization that may in any way gain or lose financially from the publication of this manuscript, either now or in the future.

None of the authors hold any stocks or shares in any organization that may in any way gain or lose financially from the publication of this manuscript, either now or in the future.

We are not applying for any patents relating to the content of this manuscript. We have also not received reimbursements, fees, funding, or salary from an organization that holds or has applied for patents relating to the content of the manuscript.

Non-financial competing interests

There are also no political, personal, religious, academic, ideological, intellectual, commercial or any other interests to declare in relation to this manuscript.

## Authors' contributions

TAS conceived of the study, and participated in its design and coordination. All authors participated in the administering of questionnaires and read and approved the final manuscript. SOS, KCE and EI wrote the first draft of the manuscript while OEO and MOM wrote the final draft with TAS.

## Pre-publication history

The pre-publication history for this paper can be accessed here:


